# Factors associated with antibiotic use during pregnancy in Sweden: a population-based cohort study

**DOI:** 10.1186/s12884-025-07736-4

**Published:** 2025-06-19

**Authors:** Aya Olivia Nakitanda, Laura Pazzagli, Björn Pasternak, Ingvild Odsbu

**Affiliations:** 1 Centre for Pharmacoepidemiology, Department of Medicine Solna, Karolinska Institutet, Eugeniahemmet T4, Astrid Cleves gata 30A, Stockholm, 17176 Sweden; 2 Clinical Epidemiology Division, Department of Medicine Solna, Karolinska Institutet, Stockholm, Sweden; 3 Department of Epidemiology Research, Statens Serum Institut, Copenhagen, Denmark; 4 Department of Chronic Diseases, Norwegian Institute of Public Health, Oslo, Norway

**Keywords:** Antibiotics, Pharmacoepidemiology, Pregnancy, Sweden

## Abstract

**Background:**

Understanding determinants of antibiotic use during pregnancy is crucial for optimizing their utilization in clinical practice. We aimed to investigate which individual-level factors are associated with antibiotic use among pregnant women.

**Methods:**

Population-based cohort study using linked data from the Swedish national health and population registers spanning from 2006 to 2019. Sociodemographic characteristics, medical and obstetric history, lifestyle and healthcare utilization were investigated as independent variables. Any systemic antibiotic use during pregnancy, the primary outcome, was determined from filled prescriptions, with broad-spectrum antibiotics and multiple courses of antibiotics as secondary outcomes. Multiple logistic regression was used to estimate odds ratios (ORs), including all independent variables in the model.

**Results:**

Among 20 variables associated with any systemic antibiotic use during pregnancy, preconception prescription drug use (OR 1.93 [95% CI 1.87–1.98] for > 4 vs. 0–1 unique therapeutic subgroups), chronic renal disease (1.82 [1.73–1.93]) and low maternal age (1.67 [1.61–1.74] for ages < 20 vs. 30–34 years) had the highest odds. Chronic renal disease, immunodeficiency disorders and preconception drug use (> 4 vs. 0–1 therapeutic subgroups) had the highest odds of broad-spectrum antibiotic use and use of multiple antibiotic courses.

**Conclusions:**

Pre-existing morbidities and low maternal age were most strongly associated with antibiotic use during pregnancy, suggesting a needs-based approach in prescribing. Proactive management of morbidities and infection prevention strategies, particularly targeting young women of reproductive age, could potentially reduce the need for antibiotic treatment in prenatal care.

**Supplementary Information:**

The online version contains supplementary material available at 10.1186/s12884-025-07736-4.

## Background

Despite ongoing initiatives to combat antibiotic resistance through optimization of antibiotic use, global antibiotic consumption is projected to continue increasing and the escalating use of broad-spectrum antibiotics also raises significant concerns about the accelerated development and spread of resistance [1]. Understanding the factors underlying these trends, particularly among population groups with high consumption, is critical to inform future strategies.

Beyond over-arching contextual factors, such as the prevailing burden of infectious diseases, regulatory frameworks, economic factors and healthcare policies [2]; the attitudes and behaviour of both prescribers and patients have been determined to ultimately shape prescribing practices, prescription filling, actual intake and eventual adherence to antibiotic therapy [3], [4]. Furthermore, socioeconomic, demographic and health-related factors have consistently been strongly associated with antibiotic use at individual level [3], with sex, age and geographical differences reported in Sweden [5], [6], [7].

However, much of this evidence is drawn from the general population and less is known specifically about pregnant women, a population in frequent need of antibiotics. Moreover, existing data lack the granularity to address the nuances of pregnancy such as maternal morbidities, elevated infection risks and prescribing decisions that may be influenced by direct safety concerns for the fetus [8], [9]. Yet, studies on pregnant women have typically assessed only a limited range of factors and scope of antibiotic use, with few studies evaluating broad-spectrum antibiotic use and potential overuse beyond filling multiple prescriptions. [10], [11], [12], [13], [14].

Notwithstanding the relatively low and consistently declining outpatient antibiotic use during pregnancy observed in Sweden in recent years [15], a better understanding of determinants of antibiotic use is critical to guide clinical risk-benefit assessments and communication, as well as to address possible treatment gaps. This study aimed to investigate the individual-level factors that are associated with antibiotic use among pregnant women who gave birth in Sweden, focusing on any systemic antibiotic use, broad-spectrum antibiotic use and the use of multiple courses of antibiotics.

## Methods

### Data sources

We performed a register-based cohort study using data from the Swedish national health and population registers, with data linkages enabled by unique personal identifiers assigned to all residents at birth or immigration. The study population was identified from the Medical Birth Register which contains antenatal and perinatal information on all pregnancies delivered in Sweden [16]. Information on morbidities registered during the first visits in antenatal care and captured in the Medical Birth Register, were supplemented with data from the National Patient Register, which contains extracts of medical records pertaining to diagnoses (International Classification of Diseases and Health Related Problems 10th revision; ICD-10) made in specialist outpatient care and during hospitalization [17]. Information relating to prescriptions filled in pharmacies across the country, with their Anatomical Therapeutic Chemical (ATC) codes and date of fill [18], were obtained from the Prescribed Drug Register [19]. The Total Population Register provided residence data by county [20], while the Longitudinal Integrated Database for Health Insurance and Labour Market Studies provided information on education [21].

### Study population

The study population included all pregnancies delivered in Sweden between 1 July 2006 and 31 December 2019, except those with missing or implausible gestational age at delivery in days (< 22 completed weeks or > 45 weeks); and/or with non-continuous residence in Sweden during the one-year period prior to pregnancy up to delivery. This resulted in 1,403,652 pregnancies from 869,700 women that met the inclusion criteria (Figure s1).

### Outcome measure

The primary outcome was any antibiotic use during pregnancy and was defined as at least one prescription for systemic antibiotics (ATC code J01 except J01XX05) filled between the last menstrual period (LMP) and the day before delivery. LMP was computed from the sonography-estimated gestational age at birth as the date of birth minus gestational age at birth in days. Gestation age is estimated around 18 weeks of gestation when at least 95% of pregnant women in Sweden undergo a routine ultrasound scan [22].

### Independent variables

We identified 25 individual-level potential prognostic factors of antibiotic use during pregnancy based on clinical relevance, and from previous studies conducted in Sweden or similar settings [5], [11], [12], [14], [15], [23]. These included a range of sociodemographic characteristics (maternal age, highest education attained, country of birth, partnership, county of residence categorised by average density of doctors in the study period); obstetric factors (parity, conception by assisted reproductive technology [ART]); body mass index (BMI); morbidities (asthma, chronic renal disease, diabetes mellitus, immunodeficiency disorders, substance use disorders including alcohol); lifestyle (smoking); healthcare utilization and prescription drug use (where > 4 unique therapeutic subgroups indicates polypharmacy); [24] as assessed before or at the start of pregnancy (Table s1). Missing values were considered a stratum for each independent variable.

### Statistical analyses

Prevalence of antibiotic use within variable strata were calculated as the percentage of deliveries in each stratum with at least one antibiotic prescription fill and presented as row percentages. The associations between independent variables of interest and antibiotic use during pregnancy were estimated using logistic regression to obtain odds ratios (ORs) and 95% confidence intervals (CIs) using two approaches. Initially, simple regression models were used to estimate crude associations between each independent variable and antibiotic use during pregnancy. This was followed by multiple logistic regression in which all independent variables under investigation were included in the model to generate ORs. The ORs for each variable obtained from multiple regression was then interpreted as the estimated proportional difference in the odds of antibiotic use compared to the reference stratum of the variable holding all other variables in the model constant. We accounted for correlations between pregnancies carried by the same mother by computing cluster-robust standard errors. All statistical analyses were performed using R [25].

### Supplementary analyses

We performed two supplementary analyses. First, we restricted the study cohort to those with no missing information on all independent variables i.e., complete case analysis, contrasting from the main analysis where a stratum for missing values was used. Secondly, owing to the variability in antibiotic use during pregnancy with increased use in the later part [15], trimester-specific analyses were performed aimed at elucidating whether factors associated with antibiotic use varied during the course of pregnancy. Trimesters were defined as LMP (day 0) to gestational day 97 (first), gestational day 98 to 202 (second), and gestational day 203 to the day before delivery (third).

### Secondary analyses

In a subpopulation of pregnancies during which at least one prescription for systemic antibiotics was filled (*N* = 293,984; Figure s1), we aimed to investigate factors associated with broad-spectrum antibiotic use and the use of multiple courses of antibiotics during pregnancy. Broad-spectrum antibiotic use during pregnancy was defined as any prescription for broad-spectrum antibiotics filled between LMP and the day before delivery. Such antibiotics included broad-spectrum penicillins, cephalosporins, macrolides and fluoroquinolones, and were adapted from an indicator that has been proposed to monitor changes in the quality of outpatient antimicrobial use at European level (Table s2) [26]. Multiple antibiotic courses was defined as at least two successive antibiotic prescriptions filled between LMP and the day before delivery, at least 14 days apart. This analysis was aimed at investigating potential recurrent infections/episodes requiring multiple treatment courses during pregnancy (rather than multiple treatments for the same infection). Statistical analyses were conducted as for the primary outcome described earlier.

## Results

### Factors associated with any systemic antibiotic use during pregnancy

At least one prescription for systemic antibiotics was filled in 293,984 (20.9%) of 1,403,652 pregnancies among 869,700 women. The majority (62.3%) of antibiotics were prescribed in primary care, with 22.4% in specialist care besides obstetrics, 14.5% in obstetric care and 0.8% in infectious disease units.

**Fig. 1:**
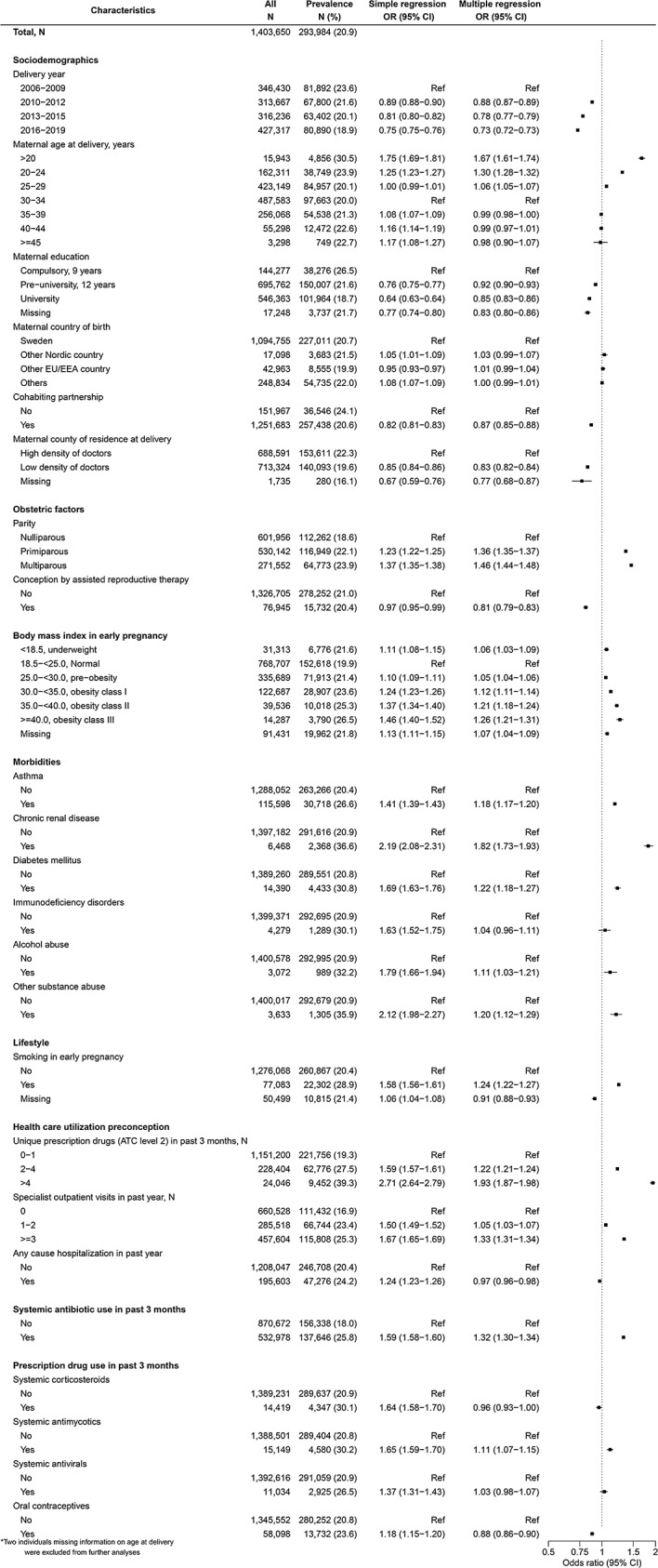
Associations between maternal characteristics and any systemic antibiotic use during pregnancy

With multiple regression, positive associations were observed for parity (vs. nulliparity), pre-existing morbidities (vs. none), higher or lower BMI (vs. normal), substance use disorders including alcohol, smoking during early pregnancy, prescription drug use (antibiotics and antimycotics), high healthcare utilization (vs. none to few), of which preconception prescription drug use (OR 1.93 [95% CI, 1.87–1.98] for > 4 vs. 0–1 unique therapeutic subgroups), chronic renal disease (1.82 [1.73–1.93]) and lower maternal age (1.67 [1.61–1.74] for ages < 20 vs. 30–34 years) were most strongly associated (Fig. 1). Inverse associations were observed by later delivery years, cohabiting partnership, residence, conception by ART, any cause hospitalization, preconception oral contraceptive use, with the strongest associations for later delivery years (0.73 [0.72–0.73] for 2016–2019 vs. 2006–2009). Maternal country of birth, immunodeficiency disorders, preconception antiviral and corticosteroid use were not associated with antibiotic use during pregnancy in the multiple regression model.

The results of the complete case analysis were similar to those of the main analysis (Table s3, Table s4a). Compared to the overall analyses for the duration of the whole pregnancy, the patterns in prevalence across variable strata as well as the measures of association from the simple and multiple regression by trimester were largely similar (Table s4b). The exceptional differences were with the positive association for mothers born outside of the European Union or the European Economic Area (vs. Swedish-born) for first trimester use (1.11 [1.09–1.13]) and inverse associations in the second (0.96 [0.94–0.97]) and third trimesters (0.96 [0.94–0.98]); as well as no associations with alcohol use disorders with antibiotic use across all trimesters. Furthermore, positive associations were noted for immunodeficiency disorders with second trimester use (1.14 [1.04–1.24]); and any cause hospitalization with third trimester use (1.06 [1.04–1.08]).

### Factors associated with broad-spectrum antibiotic use during pregnancy

**Fig. 2:**
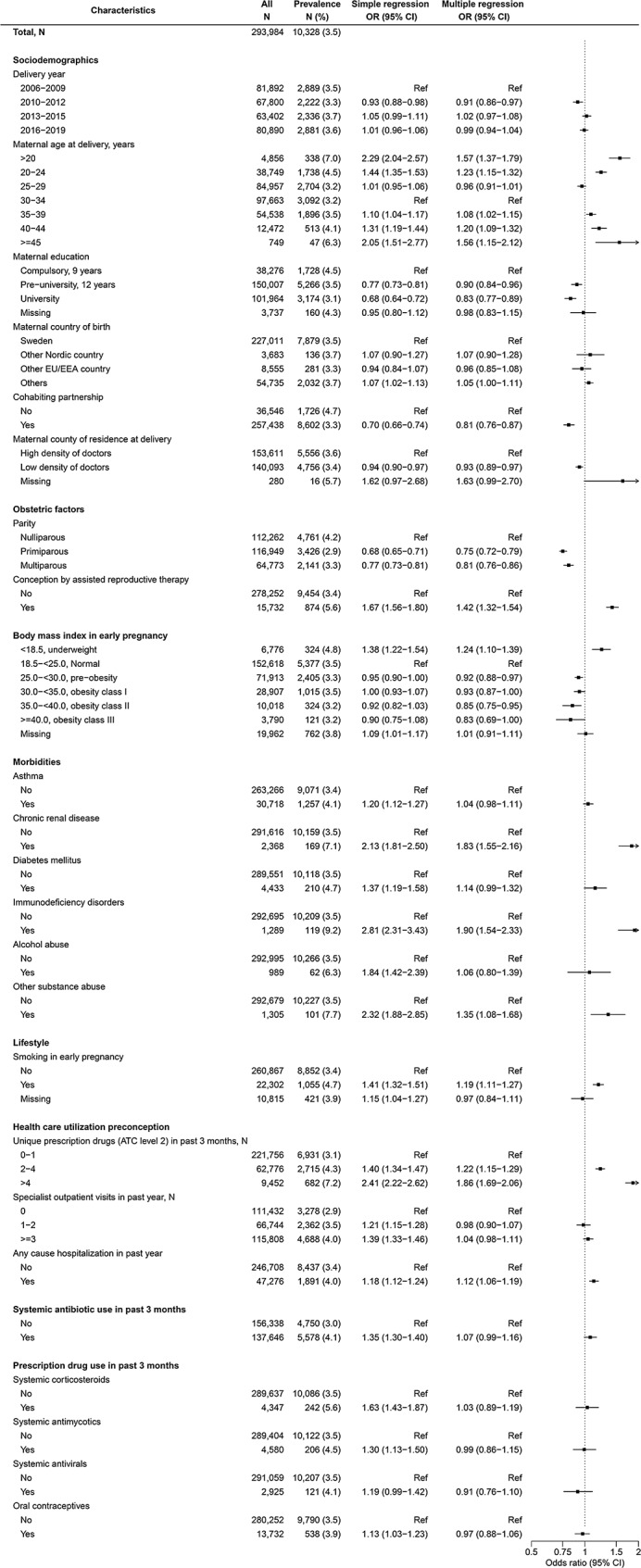
Associations between maternal characteristics and broad-spectrum antibiotic use during pregnancy

Among 293,984 pregnancies during which at least one antibiotic prescription was filled, 10,328 (3.5%) filled prescriptions for broad-spectrum antibiotics (Fig. 2). Most (9,152 [88.6%]) filled only one prescription for a broad-spectrum antibiotic. Of the total 12,310 prescriptions filled in 10,328 pregnancies, ciprofloxacin (28.0%), ceftibuten (23.9%), amoxicillin with beta-lactamase inhibitor (16.6%) and azithromycin (15.9%) were the most frequent broad-spectrum antibiotic substances. About 40% of broad-spectrum antibiotics were prescribed in specialist care outside obstetrics, while 34.1%, 20.2% and 5.8% were prescribed in primary care, obstetric and infectious disease units, respectively.

While multiple regression analyses yielded positive associations for maternal age (lower and higher vs. 30–34 years), parity (vs. nulliparity), conception by ART, chronic renal disease, immunodeficiency disorders, substance use disorders, smoking, higher number of preconception prescription drug subgroups, any cause hospitalization, and lower BMI/underweight (vs. normal); inverse associations were observed for higher education level, cohabiting partnerships, low (vs. high) density of doctors in the county of residence, parity (vs. nulliparity) and higher BMI (vs. normal). Of these, immunodeficiency disorders (OR 1.90 [95% CI, 1.54–2.33]), preconception prescription drug use (1.86 [1.69–2.06] for > 4 vs. 0–1 unique therapeutic subgroups) and chronic renal disease (1.83 [1.55–2.16]) demonstrated the strongest positive associations. For pregnancies conceived by ART, the association with broad-spectrum antibiotic use was driven by prevalent use of ciprofloxacin (23.7%) and azithromycin (22.4%) during the first trimester. The strongest inverse associations were observed for parity (0.75 [0.72–0.79], 0.81 [0.76–0.86] for primiparity, multiparity vs. nulliparity, respectively). Delivery year, maternal country of birth, asthma, diabetes mellitus, alcohol use disorders, specialist outpatient care visits and preconception prescription drug use were not associated with broad spectrum antibiotic use during pregnancy.

### Factors associated with use of multiple antibiotic courses during pregnancy

**Fig. 3:**
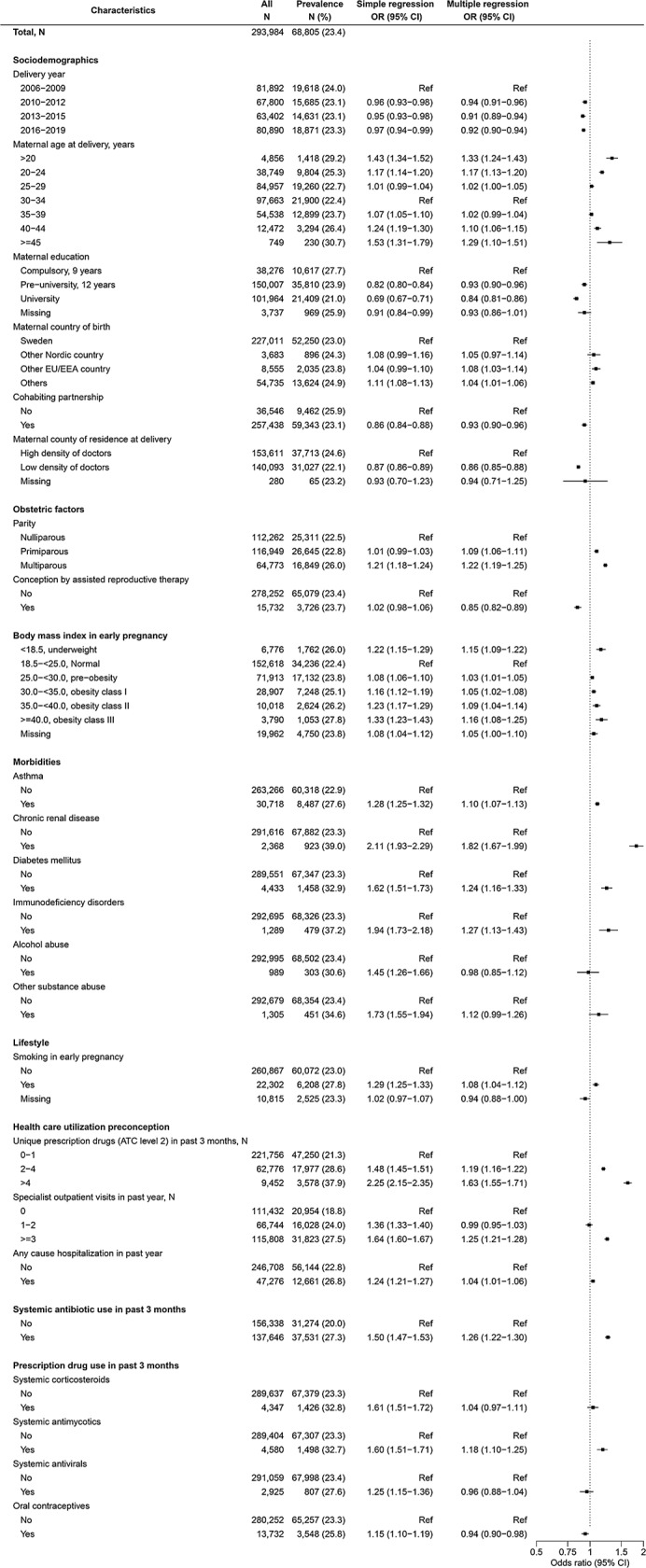
Associations between maternal characteristics and use of multiple antibiotic courses during pregnancy

Multiple courses of antibiotics were dispensed in 68,805 (23.4%) of 293,984 pregnancies (Fig. 3), among which 48,295 (70.2%) and 13,380 (19.4%) received two and three courses, respectively. From multiple regression, factors with the strongest positive associations were chronic renal disease OR 1.82 [95% CI, 1.67–1.99] and preconception prescription drug use (1.63 [1.55–1.71] for > 4 vs. 0–1 unique therapeutic subgroups); while maternal age (lower and higher vs. 30–34 years), higher parity, higher or lower BMI, other morbidities, smoking, higher healthcare utilization, preconception antibiotics and antimycotics use were positively associated to a less extent. Those with the strongest inverse association was higher maternal education (0.84 [0.81–0.86] for university level vs. compulsory education); but delivery year, cohabiting partnership, county of residence, conception by ART and preconception oral contraceptive use were also inversely associated with use of multiple courses of antibiotics during pregnancy. Maternal country of birth, substance use disorders (including alcohol), preconception corticosteroid or antiviral use were not associated with use of multiple antibiotic courses during pregnancy.

## Discussion

This large population-based cohort study identified several sociodemographic, obstetric, morbidities, lifestyle, healthcare utilization and prescription drug use characteristics of women who gave birth in Sweden associated with antibiotic use during pregnancy. While the magnitude of association was modest for the majority of these characteristics when including all factors of interest in the multiple regression analyses, pre-existing morbidities, preconception polypharmacy and maternal age below 20 years were most strongly associated with antibiotic use overall. These key factors were consistent for both use of broad-spectrum antibiotics and multiple courses of antibiotics during pregnancy. Findings from both complete case analysis and analyses by trimester were largely aligned with the main findings, with the latter providing insights into drivers of antibiotic use at different stages of gestation. It can be anticipated that young women and women with pre-existing chronic renal disease are most likely to fill prescriptions for antibiotics when pregnant, including broad-spectrum antibiotics and multiple courses of antibiotics.

Our findings align closely with similar studies from Sweden and other European countries, yet expand on the range of potential factors previously evaluated. Building on a prior utilization study [15], the declining trends of antibiotic use in pregnancy were confirmed using calendar year as a factor in the multiple regression analyses [15], and additionally showed that the vast majority of prescriptions for systemic antibiotics were issued in primary care settings. Socio-demographically, antibiotic use during pregnancy showed differences driven by maternal age, education and cohabiting partnerships; but differences by country of birth were less apparent, consistent with observations from the Swedish general population [5], [23]. In this regard, previous studies from Denmark and Norway also found that a higher level of education, cohabiting partnership and maternal age were inversely associated with antibiotic use, with the highest prevalence of use among pregnant women aged less than 24 years [11], [12]. Geographically, the odds of prenatal antibiotic use were significantly lower in counties with fewer compared with a higher number of physicians per capita (Uppsala, Västerbotten, Stockholm, Skåne and Östergötland). A similar association related to physician density was observed between the German federal states [10].

Parity was linked to a higher tendency to fill antibiotic prescriptions in the current study, in line with multiple similar studies undertaken in Europe [11], [12], [14]. However, contrary to findings in France and Norway, fertility treatment was associated with lower odds of antibiotic use during pregnancy in this study [12], [14]. This divergence may arise from differences in clinical guidelines, wherein prophylactic antibiotics may be routinely given prior to embryo transfer to patients undergoing in-vitro fertilization treatments in some countries, unlike in Sweden [27]. Also, it is plausible that women who sought fertility treatment may adopt a more health-conscious lifestyle (in terms such as hygiene and nutrition) to enhance favourable pregnancy outcomes, that may ultimately contribute to mitigating common infections. Maternal BMI and smoking status in early pregnancy demonstrated prognostic patterns in our study akin to Denmark, despite the Danish study only presenting bivariable associations [11]. Smoking is a known risk factor for infections particularly of the respiratory tract [28], while high BMI, mediated by gestational diabetes, raises risk of infections consequently [29]. Among the selected prescription drug use assessed preconception, antibiotics emerged with the highest odds of prenatal antibiotic use. This corroborates findings from France, and likely reflects an underlying susceptibility to infections necessitating antibiotic therapy [14]. Although we hypothesized that women who were on oral contraceptives prior to conception may be at higher risk of infections from the direct effects of the drug and potentially increased sexual activity [30], 12% lower odds were observed.

Pre-existing chronic renal disease, alongside preconception polypharmacy demonstrated the highest positive association of antibiotic use during pregnancy with close to two-fold higher odds. Preconception prescription drug use and specialist outpatient visits, used as measures of healthcare utilization, also reflect underlying morbidities; and as such polypharmacy likely represents multimorbidity. Although asthma and pre-existing diabetes were associated to a less degree, similar associations have been reported in other countries for asthma and as summary indices for multimorbidities [11, 12, 14, 31]. Strong associations between recurrent urinary tract infections and antibiotic use during pregnancy have been demonstrated in Norway [12], and the observed strong association with chronic renal disease in this study could, in part, be explained by this. It may, at the same time, reflect a surveillance bias as pregnant women with pre-existing renal disease receive specialist care owing to the risk of a range of maternal morbidities and consequently adverse perinatal outcomes [32]. Chronic renal disease is, nevertheless, a major risk factor for infections [33], and this was evident in our results as it was strongly associated not only antibiotic use in general but also of broad-spectrum antibiotics and multiple courses of antibiotics during pregnancy consistently.

Broad-spectrum antibiotic use during pregnancy in Sweden was stably low over the study period, and this was in line with the relatively low use reported at population level compared to other European countries [34]. Moreover, the proportion of pregnant antibiotic users who were dispensed broad-spectrum antibiotics in Sweden was also lower than reported in Norway (4.6%), where fluoroquinolones were not considered a broad-spectrum antibiotic [12]. The limited use and choice of broad-spectrum antibiotics could be explained by adherence to clinical guidelines for the treatment of common infections by prescribers, coupled with low resistance rates in the country, prompting use only where particularly warranted. In contrast to overall antibiotic use, most prescriptions for broad-spectrum antibiotics were issued in specialist care outside of obstetrics and the direction of associations differed for several factors. Notably, immunodeficiency disorders were most strongly associated with broad-spectrum antibiotic use, with close to two-fold increased odds. These further indicate their reserved use for less common, atypical or severe infections, that are not managed in primary care and whose risk factors may also differ from common infections. Exceptionally, conception by ART that was inversely associated with any antibiotic use during pregnancy, was positively associated with broad-spectrum antibiotic use driven by prevalent use of ciprofloxacin and azithromycin during the first trimester.

The present study is, to our knowledge, the first to explore use of multiple antibiotic courses during pregnancy, and found that pregnant women with chronic renal disease not only had the highest odds of any systemic antibiotic and broad-spectrum antibiotic use, but were also most susceptible to using multiple antibiotic courses with close to two-fold higher odds. Nearly a quarter of pregnant antibiotic users received multiple courses but the vast majority did not receive more than three courses. The prevalence of multiple courses remained stable during the study period. The patterns of association between the factors investigated and multiple antibiotic courses, in contrast to broad-spectrum antibiotics, more closely aligned with the overall use.

### Strengths and limitations

Alongside several strengths, our study has limitations. The present study utilized data from high-quality registers with nationwide coverage and investigated associations of numerous independent variables with antibiotic use during pregnancy. However, our analyses were limited to deliveries after 22 completed weeks (28 weeks before July 2008) of gestation, effectively excluding abortions; and to those variables available in our datasets, missing potentially relevant information such as occupation and income level [5], [16]. The datasets did not contain information on the indication for which antibiotics were prescribed, which limited our interpretation of the results as relates to the appropriateness of antibiotics prescribed. We also acknowledge potential misclassification of the outcome which could have led to biased estimates of antibiotic use during pregnancy overall and by trimester, as neither actual intake of the dispensed antibiotics, the timing of intake nor antibiotics administered in hospitals were ascertained. In the supplementary and secondary analyses, part of the differences in associations with that observed in the main analyses may have arisen from selection bias, reduction in variable strata and hence statistical power. This may have affected variables with particularly low baseline prevalence in the population such as alcohol use disorders. Broad-spectrum antibiotics covered in this study were limited to those prone to overuse in ambulatory care [26]. Given that our findings are influenced by the prevailing antibiotic prescribing practices in Sweden, their generalizability to settings characterized by widespread antibiotic misuse and overuse is limited. Furthermore, even after a careful consideration of the potential factors to be included, model specification due to variable selection may have affected the findings from the study. Despite some missing values for key variables such as BMI and smoking in early pregnancy, the results from the complete case analysis showed alignment with those from the main analysis.

## Conclusions

Pre-existing maternal morbidities and maternal age below 20 years were most strongly associated with antibiotic use during pregnancy in Sweden, among which pregnant women with chronic kidney disease were most prone to use antibiotics, including broad-spectrum antibiotics and multiple antibiotic courses. Antibiotic use during pregnancy in Sweden appears driven by legitimate medical need arising from the frequent common infections and underlying susceptibility, and a needs-based clinical approach thereof, rather than being indicative of misuse or overuse. Proactive management of pre-existing morbidities and infection prevention strategies, particularly targeting young women in reproductive age, could potentially reduce the need for antibiotic treatment in prenatal care.

## Electronic supplementary material

Below is the link to the electronic supplementary material.

Supplementary Material 1

## Data Availability

The data that support the findings of this study are sensitive personal information that cannot be made available as mandated by the EU General Data Protection Regulation (GDPR), the Swedish Data Protection Act and the Swedish Act on Health Data Registers.

## Abbreviations

ART: Assisted reproductive technology
ATC: Anatomic therapeutic chemical
BMI: Body mass index
CI: Confidence interval
LMP: Last menstrual period
OR: Odds ratio
