# Supplementary material for: Factors associated with antibiotic use during pregnancy in Sweden: a population-based cohort study
Source: BMC Pregnancy Childbirth. 2025 Jun 19;25:672. doi: 10.1186/s12884-025-07736-4 (PMC12180237; doi:10.1186/s12884-025-07736-4)
Supplement: Supplementary file 1 — Supplementary Material 1 [file 12884_2025_7736_MOESM1_ESM.docx]

**Supplementary material**

Nakitanda AO, Pazzagli L, Pasternak B, Odsbu I.

Factors associated with antibiotic use during pregnancy in Sweden: a population-based cohort study

Figure s1. Flowchart depicting cohort selection

Table s1. Definition and assessment window of predictors (independent variables) under investigation

Table s2. Definition of broad-spectrum antibiotics

Table s3. Baseline characteristics of the study population for the main analysis and supplementary analysis by trimester (all pregnancies); secondary analyses for broad spectrum antibiotic use and use of multiple antibiotic courses (any systemic antibiotic prescription filled); and complete case analysis

Table s4. Supplementary analyses on associations between maternal characteristics and any systemic antibiotic use during pregnancy (a) Complete case analysis (b) Antibiotic use by trimester

**Figure s1. Flowchart depicting cohort selection**

Excluded:

321 Missing, implausible gestational age at birth

80,226 Non-continuous maternal residence in Sweden during the one-year period pre-conception until delivery

(3 deliveries fulfilled both criteria)

Eligible pregnancies

N= 1,403,652

(From 869,700 women)

Any prescription fills for systemic antibiotics during pregnancy

N= 293,984

All pregnancies delivered in Sweden

1 July 2006 – 31 December 2019

N= 1,484,196

**Table s1.** **Definition and assessment window of predictors (independent variables) under investigation**

|  | Assessment window | Definition | Categories | Source |
| --- | --- | --- | --- | --- |
| **Demographics** |  |  |  |  |
| Calendar year of delivery |  | Birth record | 2006-2009, 2010-2012,2013-2015, 2016-2019 | Medical Birth Register |
| Maternal age at delivery |  | Birth record | <20, 20-<25,25-<30, 30-<35, 35-<40, 40-<45, ≥45 | Medical Birth Register |
| Maternal education level in delivery year |  | Highest attained education | Compulsory, Pre-university, University, missing | LISA |
| Maternal country of birth |  | Antenatal record | Sweden, other Nordic country, other EU country, others, missing | Medical Birth Register |
| Cohabiting partnership |  | Antenatal record | Yes/No | Medical Birth Register |
| Maternal county of residence in delivery year |  | Most recent population record | County, categorised as high or low density of doctors | Total Population Register |
|  |  |  |  |  |
| **Obstetric history** |  |  |  |  |
| Parity |  | Antenatal record | Nulliparous, Primiparous, multiparous | Medical Birth Register |
| Conception through ART | Early pregnancy | Antenatal record | Yes/No | Medical Birth Register |
|  |  |  |  |  |
| Body mass index | Early pregnancy | Derived from antenatal weight and height measures | <18.5, 18.5-<25, 25-<30, 30-<35, 35-<40, ≥40, missing | Medical Birth Register |
|  |  |  |  |  |
| **Morbidities** | LMP-365 to LMP, LMP-90 to LMP for prescriptions |  |  |  |
| Asthma |  | Antenatal record and/or ICD-10 codes as main or other diagnoses: J45-J46 and/or ATC codes: R03AC, R03AK, R03BA, R03DC | Yes/No | Medical Birth Register, National Patient Register,  Prescribed Drug Register |
| Chronic renal disease |  | Antenatal record and/or ICD-10 codes as main or other diagnoses: N18 | Yes/No | National Patient register |
| Pre-existing diabetes |  | Antenatal record and/or ICD-10 codes as main or other diagnoses: E10-14, O24.0, O24.1, O24.2, O24.3 and/or ATC codes A10 without N97 and/or E28.2 | Yes/No | Medical Birth Register, National Patient Register,  Prescribed Drug Register |
| Immunodeficiency disorders and immunosuppressant therapy |  | ICD-10 codes as main or other diagnoses: D70-D71, D80-D84, D89 and ATC codes: L01, L04 | Yes/No | National Patient register, Prescribed Drug Register |
| Alcohol use disorder | LMP-365 to LMP+97 | ICD-10 codes: Z71.4, O35.4, F10 | Yes/No | National Patient Register |
| Other substance use disorder | LMP-365 to LMP+97 | ICD-10 codes: F11-19, Z71.5, Z86.4 | Yes/No | National Patient Register |
|  |  |  |  |  |
| **Lifestyle** |  |  |  |  |
| Smoking during early pregnancy | Early pregnancy | Antenatal record | Yes/No/missing | Medical Birth Register |
|  |  |  |  |  |
| **Maternal healthcare utilization** |  |  |  |  |
| Prescription drugs dispensed | LMP-90 to LMP | Number of unique ATC codes (2nd level) | 0-1, 2-4, ≥5 | Prescribed Drug register |
| Specialist outpatient visits | LMP-365 to LMP | Number of visits | 0, 1-2, ≥3 | National Patient register |
| Hospital admissions | LMP-365 to LMP | Any cause hospital admission | Yes/No | National Patient register |
|  |  |  |  |  |
| **History of bacterial infections** |  |  |  |  |
| Antibiotic use | LMP-90 to LMP | ATC codes: J01 excluding J01XX05 | Number of prescription fills: 0, 1-2, >2 | Prescribed Drug register |
|  |  |  |  |  |
| **Other prescription drug use** | LMP-90 to LMP | ATC codes |  | Prescribed Drug Register |
| Systemic corticosteroids |  | H02 | Yes/No |  |
| Systemic antimycotics |  | J02 | Yes/No |  |
| Systemic antivirals |  | J05 | Yes/No |  |
| Oral contraceptives |  | G03A, G03F | Yes/No |  |
|  |  |  |  |  |

**Table s2: Broad-spectrum antibiotics**

|  | Class | ATC codes |
| --- | --- | --- |
| Broad-spectrum penicillins | Combinations of penicillins, including beta-lactamase inhibitors | J01CR |
| Broad-spectrum cephalosporins | Second generation cephalosporins | J01DC |
| Broad-spectrum cephalosporins | Third generation cephalosporins | J01DD |
| Macrolides, excluding erythromycin |  | J01FA excluding J01FA01 |
| Fluoroquinolones |  | J01MA |
|  |  |  |

*ATC Anatomic Therapeutic Chemical*

**Table s3.** **Baseline characteristics of the study population for the main analysis and supplementary analysis by trimester (all pregnancies), secondary analyses for broad spectrum antibiotic use and use of multiple antibiotic courses (any systemic antibiotic prescription filled) and complete case analysis**

|  | All pregnancies  N (%) | Any systemic antibiotic prescription filled  N (%) | Complete cases  N (%) |
| --- | --- | --- | --- |
|  |  |  |  |
| Total | 1,403,652 | 293,984 | 1,289,905 |
|  |  |  |  |
| Sociodemographics |  |  |  |
| Delivery year |  |  |  |
| 2006-2009 | 346,430 (24.7) | 81,892 (27.9) | 311,487 (24.1) |
| 2012-2010 | 313,668 (22.3) | 67,800 (23.1) | 288,534 (22.4) |
| 2013-2015 | 316,236 (22.5) | 63,402 (21.6) | 293,626 (22.8) |
| 2016-2019 | 427,318 (30.4) | 80,890 (27.5) | 396,258 (30.7) |
| Maternal age at delivery, years |  |  |  |
| <20 | 15,943 (1.1) | 4,856 (1.7) | 13,245 (1.0) |
| 20-24 | 162,311 (11.6) | 38,749 (13.2) | 147,033 (11.4) |
| 25-29 | 423,149 (30.1) | 84,957 (28.9) | 390,758 (30.3) |
| 30-34 | 487,583 (34.7) | 97,663 (33.2) | 449,864 (34.9) |
| 35-39 | 256,068 (18.2) | 54,538 (18.6) | 235,455 (18.3) |
| 40-44 | 55,298 (3.9) | 12,472 (4.2) | 50,608 (3.9) |
| ≥45 | 3,298 (0.2) | 749 (0.3) | 2,942 (0.2) |
| Missing | 2 (<0.1) | 0(0), | - |
| Highest education attained by delivery year |  |  |  |
| Compulsory, 9 years | 144,279 (10.3) | 38,276 (13.0) | 133,841 (10.4) |
| Pre-university, 12 years | 695,762 (49.6) | 150,007 (51.0) | 647,805 (50.2) |
| University | 546,363 (38.9) | 101,964 (34.7) | 508,259 (39.4) |
| Missing | 17,248 (1.2) | 3,737 (1.3) | - |
| Maternal country of birth |  |  |  |
| Sweden | 1,094,757 (78) | 227,011 (77.2) | 1,013,905 (78.6) |
| Other Nordic country | 17,098 (1.2) | 3,683 (1.3) | 14,513 (1.1) |
| Other EU/EEA country | 42,963 (3.1) | 8,555 (2.9) | 38,224 (3.0) |
| Others | 248,834 (17.7) | 54,735 (18.6) | 223,263 (17.3) |
| Cohabiting partnership at delivery | 1,251,684 (89.2) | 257,438 (87.6) | 1,193,901 (92.6) |
| Maternal county of residence at delivery year |  |  |  |
| High density of doctors | 688,591 (49.1) | 153,611 (52.3) | 639,430 (49.6) |
| Low density of doctors | 713,326 (50.8) | 140,093 (47.7) | 650,475 (50.4) |
| Missing | 1,735 (0.1) | 280 (0.1) | - |
|  |  |  |  |
| Obstetric factors |  |  |  |
| Parity |  |  |  |
| Nulliparous | 601,957 (42.9) | 112,262 (38.2) | 554,061 (43.0) |
| Primiparous | 530,143 (37.8) | 116,949 (39.8) | 489,228 (37.9) |
| Multiparous | 271,552 (19.3) | 64,773 (22.0) | 246,616 (19.1) |
| Conception by assisted reproductive technology | 76,945 (5.5) | 15,732 (5.4) | ,73,953 (5.7) |
|  |  |  |  |
| Body mass index |  |  |  |
| <18.5, underweight | 31,313 (2.2) | 6,776 (2.3) | 30,515 (2.4) |
| 18.5 – <25.0, normal | 768,708 (54.8) | 152,618 (51.9) | 757,491 (58.7) |
| 25.0 – <30.0, pre-obesity | 335,690 (23.9) | 71,913 (24.5) | 329,471 (25.5) |
| 30.0 – <35.0, obesity class I | 122,687 (8.7) | 28,907 (9.8) | 119,871 (9.3) |
| 35.0 – <40.0, obesity class II | 39,536 (2.8) | 10,018 (3.4) | 38,595 (3.0) |
| ≥40.0, obesity class III | 14,287 (1.0) | 3,790 (1.3) | 13,962 (1.1) |
| Missing | 91,431 (6.5) | 19,962 (6.8) | - |
|  |  |  |  |
| Morbidities |  |  |  |
| Asthma | 115,598 (8.2) | 30,718 (10.4) | 109,198 (8.5) |
| Chronic renal disease | 6,468 (0.5) | 2,368 (0.8) | 6,108 (0.5) |
| Pre-existing diabetes mellitus | 14,390 (1.0) | 4,433 (1.5) | 13,338 (1.0) |
| Immunodeficiency disorders | 4,279 (0.3) | 1,289 (0.4) | 3,950 (0.3) |
| Alcohol use disorder | 3,072 (0.2) | 989 (0.3) | 2,774 (0.2) |
| Substance use disorder | 3,633 (0.3) | 1,305 (0.4) | 3,225 (0.3) |
|  |  |  |  |
| Lifestyle |  |  |  |
| Smoking in early pregnancy |  |  |  |
| No | 1,276,070 (90.9) | 260,867 (88.7) | 1,217,061 (94.4) |
| Yes | 77,083 (5.5) | 22,302 (7.6) | 72,844 (5.6) |
| Missing | 50,499 (3.6) | 10,815 (3.7) | - |
|  |  |  |  |
| Healthcare utilization preconception |  |  |  |
| Unique prescription drugs (ATC level 2), N |  |  |  |
| 0-1 | 1,151,202 (82.0) | 221,756 (75.4) | 1,058,093 (82.0) |
| 2-4 | 228,404 (16.3) | 62,776 (21.4) | 209,842 (16.3) |
| ≥5 | 24,046 (1.7) | 9,452 (3.2) | 21,970 (1.7) |
| Specialist outpatient visits |  |  |  |
| 0 | 660,530 (47.1) | 111,432 (37.9) | 606,557 (47) |
| 1-2 | 285,518 (20.3) | 66,744 (22.7) | 262,414 (20.3) |
| ≥3 | 457,604 (32.6) | 115,808 (39.4) | 420,934 (32.6) |
| Hospitalization | 195,603 (13.9) | 47,276 (16.1) | 177,115 (13.7) |
|  |  |  |  |
| Maternal bacterial infections preconception |  |  |  |
| Systemic antibiotic use | 532,978 (38.0) | 137,646 (46.8) | 490,244 (38.0) |
|  |  |  |  |
| Maternal prescription drug use preconception |  |  |  |
| Systemic corticosteroids | 14,419 (1.0) | 4,347 (1.5) | 13,264 (1.0) |
| Systemic antimycotics | 15,149 (1.1) | 4,580 (1.6) | 13,924 (1.1) |
| Systemic antivirals | 11,034 (0.8) | 2,925 (1.0) | 10,089 (0.8) |
| Oral contraceptives | 58,098 (4.1) | 13,732 (4.7) | 53,560 (4.2) |
|  |  |  |  |

**Table s4. Supplementary analyses on associations between maternal characteristics and any systemic antibiotic use during pregnancy (a) Complete case analysis**

|  | Prevalence  N (%) | Simple  regression  OR (95% CI) | Multiple regression  OR (95% CI) |
| --- | --- | --- | --- |
|  |  |  |  |
| Sociodemographics |  |  |  |
| Delivery year |  |  |  |
| 2006-2009 | 73,276 (23.5) | Ref | Ref |
| 2010-2012 | 62,240 (21.6) | 0.89 (0.88-0.90) | 0.88 (0.86-0.89) |
| 2013-2015 | 58,813 (20.0) | 0.81 (0.80-0.82) | 0.78 (0.77-0.79) |
| 2015-2019 | 74,873 (18.9) | 0.76 (0.75-0.77) | 0.72 (0.72-0.73) |
| Maternal age at delivery, years |  |  |  |
| <20 | 4,093 (30.9) | 1.79 (1.73-1.86) | 1.68 (1.61-1.75) |
| 20-24 | 35,073 (23.9) | 1.26 (1.24-1.27) | 1.31 (1.29-1.33) |
| 25-29 | 78,206 (20.0) | 1.00 (0.99-1.01) | 1.06 (1.05-1.07) |
| 30-34 | 89,809 (20.0) | Ref | Ref |
| 35-39 | 49,987 (21.2) | 1.08 (1.07-1.09) | 0.99 (0.97-1.00) |
| 40-44 | 11,377 (22.5) | 1.16 (1.14-1.19) | 0.99 (0.97-1.01) |
| ≥45 | 657 (22.3) | 1.15 (1.06-1.26) | 0.96 (0.88-1.05) |
| Highest education attained by delivery year |  |  |  |
| Compulsory, 9 years | 35,414 (26.5) | Ref | Ref |
| Pre-university, 12 years | 139,275 (21.5) | 0.76 (0.75-0.77) | 0.92 (0.91-0.94) |
| University | 94,513 (18.6) | 0.63 (0.63-0.64) | 0.85 (0.84-0.87) |
| Maternal country of birth |  |  |  |
| Sweden | 209,335 (20.6) | Ref | Ref |
| Other Nordic country | 3,126 (21.5) | 1.06 (1.01-1.10) | 1.02 (0.98-1.07) |
| Other EU/EEA country | 7,656 (20.0) | 0.96 (0.94-0.99) | 1.02 (0.99-1.05) |
| Others | 49,085 (22.0) | 1.08 (1.07-1.10) | 1.00 (0.99-1.02) |
| Cohabiting partnership |  |  |  |
| Yes | 24,309 (25.3) | Ref | Ref |
| No | 244,893 (20.5) | 0.76 (0.75-0.77) | 0.86 (0.84-0.87) |
| Maternal county of residence at delivery |  |  |  |
| High density of doctors | 142,173 (22.2) | Ref | Ref |
| Low density of doctors | 127,029 (19.5) | 0.85 (0.84-0.86) | 0.82 (0.82-0.83) |
|  |  |  |  |
| Obstetric factors |  |  |  |
| Parity |  |  |  |
| Nulliparous | 102,893 (18.6) | Ref | Ref |
| Primiparous | 107,616 (22.0) | 1.24 (1.23-1.25) | 1.36 (1.35-1.38) |
| Multiparous | 58,693 (23.8) | 1.37 (1.35-1.39) | 1.46 (1.44-1.48) |
| Conception by assisted reproductive technology |  |  |  |
| No | 254,095 (20.9) | Ref | Ref |
| Yes | 15,107 (20.4) | 0.97 (0.95-0.99) | 0.81 (0.79-0.83) |
|  |  |  |  |
| Body mass index |  |  |  |
| <18.5, underweight | 6,598 (21.6) | 1.11 (1.08-1.15) | 1.06 (1.03-1.09) |
| 18.5 – <25.0, normal | 150,322 (19.8) | Ref | Ref |
| 25.0 – <30.0, pre-obesity | 70,584 (21.4) | 1.1 (1.09-1.11) | 1.05 (1.04-1.06) |
| 30.0 – <35.0, obesity class I | 28,235 (23.6) | 1.24 (1.23-1.26) | 1.12 (1.11-1.14) |
| 35.0 – <40.0, obesity class II | 9,774 (25.3) | 1.37 (1.34-1.40) | 1.21 (1.18-1.24) |
| ≥40.0, obesity class III | 3,689 (26.4) | 1.45 (1.39-1.51) | 1.25 (1.20-1.30) |
|  |  |  |  |
| Morbidities |  |  |  |
| Asthma |  |  |  |
| No | 240,288 (20.4) | Ref | Ref |
| Yes | 28,914 (26.5) | 1.41 (1.39-1.43) | 1.19 (1.17-1.20) |
| Chronic renal disease |  |  |  |
| No | 266,966 (20.8) | Ref | Ref |
| Yes | 2,236 (36.6) | 2.20 (2.08-2.32) | 1.83 (1.73-1.94) |
| Pre-existing diabetes mellitus |  |  |  |
| No | 265,127 (20.8) | Ref | Ref |
| Yes | 4,075 (30.6) | 1.68 (1.61-1.75) | 1.21 (1.16-1.26) |
| Immunodeficiency disorders |  |  |  |
| No | 268,014 (20.8) | Ref | Ref |
| Yes | 1,188 (30.1) | 1.63 (1.52-1.76) | 1.04 (0.97-1.12) |
| Alcohol use disorder |  |  |  |
| No | 268,316 (20.8) | Ref | Ref |
| Yes | 886 (31.9) | 1.78 (1.64-1.93) | 1.11 (1.01-1.20) |
| Substance use disorder (except alcohol) |  |  |  |
| No | 268,052 (20.8) | Ref | Ref |
| Yes | 1,150 (35.7) | 2.11 (1.96-2.27) | 1.19 (1.10-1.28) |
|  |  |  |  |
| Lifestyle |  |  |  |
| Smoking in early pregnancy |  |  |  |
| No | 248,148 (20.4) | Ref | Ref |
| Yes | 21,054 (28.9) | 1.59 (1.56-1.62) | 1.24 (1.22-1.27) |
|  |  |  |  |
| Healthcare utilization preconception |  |  |  |
| Unique prescription drugs (ATC level 2) in past 3 months, N |  |  |  |
| 0-1 | 203,089 (19.2) | Ref | Ref |
| 2-4 | 57,542 (27.4) | 1.59 (1.57-1.61) | 1.23 (1.21-1.24) |
| >4 | 8,571 (39) | 2.69 (2.62-2.77) | 1.91 (1.86-1.98) |
| Specialist outpatient visits in past year, N |  |  |  |
| 0 | 102,020 (16.8) | Ref | Ref |
| 1-2 | 61,112 (23.3) | 1.5 (1.48-1.52) | 1.05 (1.03-1.07) |
| ≥3 | 106,070 (25.2) | 1.67 (1.65-1.68) | 1.32 (1.31-1.34) |
| Any cause hospitalization in past year, N |  |  |  |
| No | 226,558 (20.4) | Ref | Ref |
| Yes | 42,644 (24.1) | 1.24 (1.23-1.26) | 0.97 (0.96-0.98) |
|  |  |  |  |
| Maternal bacterial infections preconception |  |  |  |
| Systemic antibiotic use |  |  |  |
| No | 143,096 (17.9) | Ref | Ref |
| Yes | 126,106 (25.7) | 1.59 (1.58-1.60) | 1.32 (1.3-1.34) |
|  |  |  |  |
| Prescription drug use preconception |  |  |  |
| Systemic corticosteroids |  |  |  |
| No | 265,227 (20.8) | Ref | Ref |
| Yes | 3,975 (30) | 1.63 (1.57-1.69) | 0.96 (0.92-1.00) |
| Systemic antimycotics |  |  |  |
| No | 265,011 (20.8) | Ref | Ref |
| Yes | 4,191 (30.1) | 1.64 (1.58-1.70) | 1.1 (1.06-1.15) |
| Systemic antivirals |  |  |  |
| No | 266,543 (20.8) | Ref | Ref |
| Yes | 2,659 (26.4) | 1.36 (1.30-1.42) | 1.03 (0.98-1.08) |
| Oral contraceptives |  |  |  |
| No | 256,607 (20.8) | Ref | Ref |
| Yes | 12,595 (23.5) | 1.17 (1.15-1.20) | 0.88 (0.86-0.9) |
|  |  |  |  |

**Table s4. Supplementary analyses on associations between maternal characteristics and any systemic antibiotic use during pregnancy (b) Antibiotic use by trimester**

|  | First trimester | | | Second trimester | | | Third trimester | | |
| --- | --- | --- | --- | --- | --- | --- | --- | --- | --- |
|  | Prevalence  N (%) | Simple regression  OR (95% CI) | Multiple regression  OR (95% CI) | Prevalence  N (%) | Simple regression  OR (95% CI) | Multiple regression  OR (95% CI) | Prevalence  N (%) | Simple regression  OR (95% CI) | Multiple regression  OR (95% CI) |
|  |  |  |  |  |  |  |  |  |  |
| Sociodemographics |  |  |  |  |  |  |  |  |  |
| Delivery year |  |  |  |  |  |  |  |  |  |
| 2006-2009 | 32,858 (9.5) | Ref | Ref | 37,557 (10.8) | Ref | Ref | 28,070 (8.1) | Ref | Ref |
| 2010-2012 | 26,853 (8.6) | 0.89 (0.88-0.91) | 0.88 (0.87-0.90) | 30,841 (9.8) | 0.90 (0.88-0.91) | 0.88 (0.87-0.90) | 23,247 (7.4) | 0.91 (0.89-0.92) | 0.89 (0.88-0.91) |
| 2013-2015 | 24,868 (7.9) | 0.81 (0.80-0.83) | 0.79 (0.77-0.80) | 29,024 (9.2) | 0.83 (0.82-0.84) | 0.80 (0.79-0.82) | 21,876 (6.9) | 0.84 (0.83-0.86) | 0.81 (0.80-0.83) |
| 2016-2019 | 31,382 (7.3) | 0.76 (0.74-0.77) | 0.76 (0.74-0.77) | 37,324 (8.7) | 0.79 (0.78-0.80) | 0.76 (0.75-0.77) | 28,148 (6.6) | 0.80 (0.79-0.81) | 0.77 (0.76-0.78) |
| Maternal age at delivery, years |  |  |  |  |  |  |  |  |  |
| <20 | 2,199 (13.8) | 1.94 (1.85-2.03) | 1.59 (1.51-1.67) | 2,315 (14.5) | 1.69 (1.62-1.77) | 1.64 (1.56-1.72) | 1,577 (9.9) | 1.44 (1.37-1.52) | 1.57 (1.48-1.66) |
| 20-24 | 16,605 (10.2) | 1.38 (1.36-1.41) | 1.32 (1.29-1.35) | 17,834 (11.0) | 1.23 (1.21-1.25) | 1.29 (1.26-1.31) | 12,742 (7.9) | 1.12 (1.10-1.15) | 1.24 (1.21-1.27) |
| 25-29 | 34,194 (8.1) | 1.07 (1.05-1.08) | 1.08 (1.07-1.10) | 38,595 (9.1) | 1.00 (0.99-1.02) | 1.06 (1.04-1.07) | 28,407 (6.7) | 0.95 (0.93-0.96) | 1.02 (1.00-1.04) |
| 30-34 | 37,131 (7.6) | Ref | Ref | 44,428 (9.1) | Ref | Ref | 34,436 (7.1) | Ref | Ref |
| 35-39 | 20,686 (8.1) | 1.07 (1.05-1.09) | 0.99 (0.97-1.01) | 25,289 (9.9) | 1.09 (1.08-1.11) | 1.00 (0.98-1.02) | 19,395 (7.6) | 1.08 (1.06-1.10) | 0.98 (0.97-1.00) |
| 40-44 | 4,822 (8.7) | 1.16 (1.12-1.20) | 0.99 (0.96-1.02) | 5,947 (10.8) | 1.20 (1.17-1.24) | 1.02 (0.99-1.05) | 4,512 (8.2) | 1.17 (1.13-1.21) | 1.01 (0.97-1.04) |
| ≥45 | 324 (9.8) | 1.32 (1.18-1.48) | 1.08 (0.96-1.21) | 338 (10.3) | 1.14 (1.02-1.28) | 0.94 (0.84-1.06) | 272 (8.3) | 1.18 (1.04-1.34) | 1.02 (0.90-1.15) |
| Highest education attained by delivery year |  |  |  |  |  |  |  |  |  |
| Compulsory, 9 years | 16,405 (11.4) | Ref | Ref | 18,064 (12.5) | Ref | Ref | 13,124 (9.1) | Ref | Ref |
| Pre-university, 12 years | 60,294 (8.7) | 0.74 (0.73-0.75) | 0.91 (0.89-0.93) | 68,921 (9.9) | 0.77 (0.75-0.78) | 0.92 (0.90-0.94) | 51,141 (7.4) | 0.79 (0.78-0.81) | 0.93 (0.91-0.95) |
| University | 37,653 (6.9) | 0.58 (0.57-0.59) | 0.80 (0.78-0.82) | 46,101 (8.4) | 0.64 (0.63-0.66) | 0.85 (0.83-0.87) | 35,742 (6.5) | 0.70 (0.68-0.71) | 0.89 (0.87-0.91) |
| Missing | 1,609 (9.3) | 0.80 (0.76-0.85) | 0.86 (0.82-0.91) | 1,660 (9.6) | 0.74 (0.70-0.79) | 0.82 (0.77-0.86) | 1,334 (7.7) | 0.84 (0.79-0.89) | 0.89 (0.84-0.95) |
| Maternal country of birth |  |  |  |  |  |  |  |  |  |
| Sweden | 88,138 (8.1) | Ref | Ref | 104,585 (9.6) | Ref | Ref | 78,253 (7.2) | Ref | Ref |
| Other Nordic country | 1,427 (8.4) | 1.04 (0.98-1.10) | 1.05 (0.99-1.11) | 1,672 (9.8) | 1.03 (0.97-1.08) | 1.00 (0.95-1.06) | 1,307 (7.6) | 1.08 (1.01-1.14) | 1.02 (0.96-1.08) |
| Other EU/EEA country | 3,275 (7.6) | 0.94 (0.91-0.98) | 1.04 (1.00-1.08) | 3,907 (9.1) | 0.95 (0.91-0.98) | 1.00 (0.97-1.04) | 3,086 (7.2) | 1.01 (0.97-1.04) | 1.03 (0.99-1.07) |
| Others | 23,121 (9.3) | 1.17 (1.15-1.19) | 1.11 (1.09-1.13) | 24,582 (9.9) | 1.04 (1.02-1.05) | 0.96 (0.94-0.97) | 18,695 (7.5) | 1.06 (1.04-1.07) | 0.96 (0.94-0.98) |
| Cohabiting partnership at delivery |  |  |  |  |  |  |  |  |  |
| No | 15,581 (10.3) | Ref | Ref | 17,040 (11.2) | Ref | Ref | 12,056 (7.9) | Ref | Ref |
| Yes | 100,380 (8.0) | 0.76 (0.75-0.78) | 0.85 (0.83-0.87) | 117,706 (9.4) | 0.82 (0.81-0.84) | 0.88 (0.86-0.90) | 89,285 (7.1) | 0.89 (0.87-0.91) | 0.91 (0.89-0.93) |
| Maternal residence at delivery |  |  |  |  |  |  |  |  |  |
| High density of doctors | 58,160 (8.5) | Ref | Ref | 71,447 (10.4) | Ref | Ref | 55,630 (8.1) | Ref | Ref |
| Low density of doctors | 57,705 (8.1) | 0.95 (0.94-0.97) | 0.93 (0.92-0.95) | 63,178 (8.9) | 0.84 (0.83-0.85) | 0.82 (0.81-0.83) | 45,597 (6.4) | 0.78 (0.77-0.79) | 0.76 (0.75-0.77) |
| Missing | 96 (5.5) | 0.63 (0.52-0.78) | 0.70 (0.57-0.86) | 121 (7.0) | 0.65 (0.54-0.78) | 0.76 (0.63-0.91) | 114 (6.6) | 0.80 (0.66-0.97) | 0.92 (0.76-1.11) |
|  |  |  |  |  |  |  |  |  |  |
| Obstetric factors |  |  |  |  |  |  |  |  |  |
| Parity |  |  |  |  |  |  |  |  |  |
| Nulliparous | 48,013 (8.0) | Ref | Ref | 50,292 (8.4) | Ref | Ref | 35,123 (5.8) | Ref | Ref |
| Primiparous | 42,295 (8.0) | 1.00 (0.99-1.01) | 1.11 (1.09-1.12) | 53,977 (10.2) | 1.24 (1.23-1.26) | 1.37 (1.35-1.39) | 43,013 (8.1) | 1.43 (1.40-1.45) | 1.52 (1.5-1.54) |
| Multiparous | 25,653 (9.5) | 1.20 (1.18-1.22) | 1.26 (1.24-1.28) | 30,477 (11.2) | 1.39 (1.37-1.41) | 1.48 (1.45-1.51) | 23,205 (8.6) | 1.51 (1.48-1.53) | 1.57 (1.54-1.6) |
| Conception by assisted reproductive therapy |  |  |  |  |  |  |  |  |  |
| No | 109,737 (8.3) | Ref | Ref | 127,310 (9.6) | Ref | Ref | 96,113 (7.2) | Ref | Ref |
| Yes | 6,224 (8.1) | 0.98 (0.95-1.00) | 0.77 (0.75-0.8) | 7,436 (9.7) | 1.01 (0.98-1.03) | 0.85 (0.83-0.87) | 5,228 (6.8) | 0.93 (0.91-0.96) | 0.82 (0.80-0.85) |
|  |  |  |  |  |  |  |  |  |  |
| Body mass index |  |  |  |  |  |  |  |  |  |
| <18.5, underweight | 2,767 (8.8) | 1.15 (1.10-1.20) | 1.06 (1.02-1.11) | 3,215 (10.3) | 1.15 (1.11-1.20) | 1.10 (1.06-1.14) | 2,282 (7.3) | 1.08 (1.04-1.13) | 1.05 (1.00-1.10) |
| 18.5 – <25.0, normal | 59,807 (7.8) | Ref | Ref | 69,556 (9.1) | Ref | Ref | 51,946 (6.8) | Ref | Ref |
| 25.0 – <30.0, pre-obesity | 28,263 (8.4) | 1.09 (1.07-1.11) | 1.03 (1.01-1.04) | 32,811 (9.8) | 1.09 (1.07-1.10) | 1.03 (1.02-1.05) | 25,341 (7.6) | 1.13 (1.11-1.14) | 1.08 (1.06-1.09) |
| 30.0 – <35.0, obesity class I | 11,467 (9.4) | 1.22 (1.20-1.25) | 1.08 (1.06-1.10) | 13,380 (10.9) | 1.23 (1.21-1.26) | 1.10 (1.08-1.13) | 10,218 (8.3) | 1.25 (1.23-1.28) | 1.14 (1.12-1.17) |
| 35.0 – <40.0, obesity class II | 4,030 (10.2) | 1.35 (1.30-1.39) | 1.15 (1.11-1.19) | 4,692 (11.9) | 1.35 (1.31-1.40) | 1.18 (1.14-1.22) | 3,569 (9.0) | 1.37 (1.32-1.42) | 1.22 (1.18-1.27) |
| ≥40.0, obesity class III | 1,592 (11.1) | 1.49 (1.41-1.57) | 1.24 (1.18-1.31) | 1,796 (12.6) | 1.45 (1.37-1.52) | 1.23 (1.17-1.30) | 1,340 (9.4) | 1.43 (1.35-1.51) | 1.25 (1.18-1.33) |
| Missing | 8,035 (8.8) | 1.14 (1.11-1.17) | 1.04 (1.01-1.08) | 9,296 (10.2) | 1.14 (1.11-1 16) | 1.07 (1.04-1.10) | 6,645 (7.3) | 1.08 (1.05-1.11) | 1.07 (1.03-1.10) |
|  |  |  |  |  |  |  |  |  |  |
| Morbidities |  |  |  |  |  |  |  |  |  |
| Asthma |  |  |  |  |  |  |  |  |  |
| No | 103,348 (8.0) | Ref | Ref | 119,953 (9.3) | Ref | Ref | 90,481 (7.0) | Ref | Ref |
| Yes | 12,613 (10.9) | 1.40 (1.38-1.43) | 1.15 (1.12-1.17) | 14,793 (12.8) | 1.43 (1.40-1.46) | 1.19 (1.17-1.22) | 10,860 (9.4) | 1.37 (1.34-1.40) | 1.18 (1.15-1.2) |
| Chronic renal disease |  |  |  |  |  |  |  |  |  |
| No | 114,860 (8.2) | Ref | Ref | 133,483 (9.6) |  |  | 100,407 (7.2) | Ref | Ref |
| Yes | 1,101 (17.0) | 2.29 (2.14-2.45) | 1.87 (1.75-2.01) | 1,263 (19.5) | 2.30 (2.15-2.45) | 1.87 (1.75-2.00) | 934 (14.4) | 2.18 (2.03-2.34) | 1.8 (1.68-1.94) |
| Pre-existing diabetes mellitus |  |  |  |  |  |  |  |  |  |
| No | 113,937 (8.2) | Ref | Ref | 132,545 (9.5) | Ref | Ref | 99,782 (7.2) | Ref | Ref |
| Yes | 2,024 (14.1) | 1.83 (1.74-1.93) | 1.29 (1.23-1.36) | 2,201 (15.3) | 1.71 (1.63-1.80) | 1.23 (1.17-1.29) | 1,559 (10.8) | 1.57 (1.49-1.66) | 1.16 (1.10-1.22) |
| Immunodeficiency disorders |  |  |  |  |  |  |  |  |  |
| No | 115,380 (8.3) | Ref | Ref | 134,050 (9.6) | Ref | Ref | 100,857 (7.2) | Ref | Ref |
| Yes | 581 (13.6) | 1.75 (1.60-1.91) | 1.08 (0.98-1.18) | 696 (16.3) | 1.83 (1.68-2.00) | 1.14 (1.04-1.24) | 484 (11.3) | 1.64 (1.49-1.81) | 1.09 (0.98-1.20) |
| Alcohol use disorder |  |  |  |  |  |  |  |  |  |
| No | 115,516 (8.3) | Ref | Ref | 134,270 (9.6) | Ref | Ref | 100,989 (7.2) | Ref | Ref |
| Yes | 445 (14.5) | 1.88 (1.70-2.08) | 1.11 (1.00-1.24) | 476 (15.5) | 1.73 (1.57-1.91) | 1.05 (0.94-1.16) | 352 (11.5) | 1.67 (1.49-1.86) | 1.11 (0.99-1.25) |
| Substance use disorder |  |  |  |  |  |  |  |  |  |
| No | 115,363 (8.2) | Ref | Ref | 134,067 (9.6) | Ref | Ref | 100,885 (7.2) | Ref | Ref |
| Yes | 598 (16.5) | 2.19 (2.01-2.40) | 1.19 (1.08-1.30) | 679 (18.7) | 2.17 (1.99-2.36) | 1.21 (1.11-1.33) | 456 (12.6) | 1.85 (1.68-2.04) | 1.13 (1.02-1.25) |
|  |  |  |  |  |  |  |  |  |  |
| Lifestyle |  |  |  |  |  |  |  |  |  |
| Smoking in early pregnancy |  |  |  |  |  |  |  |  |  |
| No | 102,154 (8.0) | Ref | Ref | 118,865 (9.3) | Ref | Ref | 90,320 (7.1) | Ref | Ref |
| Yes | 9,390 (12.2) | 1.59 (1.56-1.63) | 1.21 (1.18-1.24) | 10,829 (14.1) | 1.59 (1.56-1.63) | 1.25 (1.22-1.28) | 7,524 (9.8) | 1.42 (1.38-1.46) | 1.15 (1.12-1.19) |
| Missing | 4,417 (8.8) | 1.10 (1.07-1.14) | 0.92 (0.88-0.96) | 5,052 (10.0) | 1.08 (1.05-1.12) | 0.94 (0.90-0.98) | 3,497 (6.9) | 0.98 (0.94-1.01) | 0.89 (0.84-0.93) |
|  |  |  |  |  |  |  |  |  |  |
| Healthcare utilization preconception |  |  |  |  |  |  |  |  |  |
| Unique prescription drugs (ATC level 2), N |  |  |  |  |  |  |  |  |  |
| 0-1 | 84,435 (7.3) | Ref | Ref | 99,861 (8.7) | Ref | Ref | 76,258 (6.6) | Ref | Ref |
| 2-4 | 27,034 (11.8) | 1.7 (1.67-1.72) | 1.25 (1.23-1.28) | 29,893 (13.1) | 1.59 (1.56-1.61) | 1.22 (1.20-1.24) | 21,643 (9.5) | 1.48 (1.45-1.50) | 1.18 (1.16-1.21) |
| >4 | 4,492 (18.7) | 2.9 (2.81-3.00) | 1.99 (1.91-2.06) | 4,992 (20.8) | 2.76 (2.67-2.85) | 1.90 (1.83-1.97) | 3,440 (14.3) | 2.35 (2.27-2.44) | 1.71 (1.64-1.78) |
| Specialist outpatient visits in past year, N |  |  |  |  |  |  |  |  |  |
| 0 | 40,958 (6.2) | Ref | Ref | 49,129 (7.4) | Ref | Ref | 38,009 (5.8) | Ref | Ref |
| 1-2 | 27,950 (9.8) | 1.64 (1.62-1.67) | 1.07 (1.04-1.10) | 30,033 (10.5) | 1.46 (1.44-1.49) | 1.03 (1.00-1.05) | 22,275 (7.8) | 1.39 (1.36-1.41) | 1.04 (1.01-1.07) |
| ≥3 | 47,053 (10.3) | 1.73 (1.71-1.76) | 1.31 (1.28-1.33) | 55,584 (12.2) | 1.72 (1.70-1.74) | 1.35 (1.32-1.37) | 41,057 (9.0) | 1.61 (1.59-1.64) | 1.31 (1.28-1.33) |
| Any cause hospitalization in past year, N |  |  |  | , |  |  |  |  |  |
| No | 97,925 (8.1) | Ref | Ref | 112,304 (9.3) | Ref | Ref | 83,425 (6.9) | Ref | Ref |
| Yes | 18,036 (9.2) | 1.15 (1.13-1.17) | 0.92 (0.91-0.94) | 22,442 (11.5) | 1.26 (1.25-1.28) | 0.98 (0.96-1.00) | 17,916 (9.2) | 1.36 (1.34-1.38) | 1.06 (1.04-1.08) |
|  |  |  |  |  |  |  |  |  |  |
| Bacterial infections preconception |  |  |  |  |  |  |  |  |  |
| Systemic antibiotic use |  |  |  |  |  |  |  |  |  |
| No | 57,652 (6.6) | Ref | Ref | 69,957 (8.0) | Ref | Ref | 54,026 (6.2) | Ref | Ref |
| Yes | 58,309 (10.9) | 1.73 (1.71-1.75) | 1.40 (1.37-1.43) | 64,789 (12.2) | 1.58 (1.57-1.60) | 1.30 (1.27-1.33) | 47,315 (8.9) | 1.47 (1.45-1.49) | 1.24 (1.21-1.27) |
|  |  |  |  |  |  |  |  |  |  |
| Preconception prescription drug use |  |  |  |  |  |  |  |  |  |
| Systemic corticosteroids |  |  |  |  |  |  |  |  |  |
| No | 114,073 (8.2) | Ref | Ref | 132,532 (9.5) | Ref | Ref | 99,782 (7.2) | Ref | Ref |
| Yes | 1,888 (13.1) | 1.68 (1.60-1.77) | 0.95 (0.90-1.00) | 2,214 (15.4) | 1.72 (1.64-1.80) | 1.00 (0.95-1.05) | 1,559 (10.8) | 1.57 (1.49-1.65) | 0.98 (0.93-1.04) |
| Systemic antimycotics |  |  |  |  |  |  |  |  |  |
| No | 113,889 (8.2) | Ref | Ref | 132,512 (9.5) | Ref | Ref | 99,680 (7.2) | Ref | Ref |
| Yes | 2,072 (13.7) | 1.77 (1.69-1.86) | 1.13 (1.08-1.19) | 2,234 (14.8) | 1.64 (1.57-1.72) | 1.11 (1.06-1.17) | 1,661 (11.0) | 1.59 (1.51-1.68) | 1.14 (1.09-1.21) |
| Systemic antivirals |  |  |  |  |  |  |  |  |  |
| No | 114,753 (8.2) | Ref | Ref | 133,335 (9.6) | Ref | Ref | 100,344 (7.2) | Ref | Ref |
| Yes | 1,208 (11.0) | 1.37 (1.29-1.45) | 0.97 (0.91-1.03) | 1,411 (12.8) | 1.38 (1.31-1.47) | 1.05 (0.99-1.11) | 997 (9.0) | 1.28 (1.2-1.37) | 1.02 (0.96-1.09) |
| Oral contraceptives |  |  |  |  |  |  |  |  |  |
| No | 110,217 (8.2) | Ref | Ref | 128,245 (9.5) | Ref | Ref | 96,755 (7.2) | Ref | Ref |
| Yes | 5,744 (9.9) | 1.23 (1.20-1.26) | 0.87 (0.84-0.89) | 6,501 (11.2) | 1.2 (1.16-1.23) | 0.92 (0.89-0.95) | 4,586 (7.9) | 1.11 (1.07-1.14) | 0.89 (0.86-0.92) |
|  |  |  |  |  |  |  |  |  |  |
